# Leaves of grass: focusing phenomics on maize leaf growth

**DOI:** 10.1186/s13059-015-0773-3

**Published:** 2015-09-17

**Authors:** Michael J. Scanlon

**Affiliations:** School of Integrative Plant Science, Plant Biology Section, Cornell University, Ithaca, NY 14853 USA

## Abstract

The detailed analysis of leaf growth dynamics, when coupled with transcriptomic research, can facilitate the discovery of genes required for leaf elongation.

Please see related Research article: http://www.genomebiology.com/2015/16/1/168

## Introduction

The past two decades of plant science have been increasingly dominated by new and powerful ‘-omics’ technologies. Phenomics entails analyses of the entire spectrum of phenotypic traits in an organism, and is an essential component to understanding how genotype translates into phenotype. Compared to the relative ease with which the genome and transcriptome of a plant or plant organ can be analyzed, plant phenomics is more challenging owing to the sheer abundance of plant phenotypic characters and the inherent difficulties in studying phenotypic dynamics throughout the entire plant life cycle (ontogeny).

Most plant phenomics studies to date have been limited to macroscopic imaging of seedling stage or mature-stage phenotypes [1]. X-ray technologies, such as computational tomography, can reveal stunning phenotypic details of internal plant structures within intact samples, but these studies are not easily adapted to high-throughput phenotyping. These technological limitations to high-throughput plant phenotyping are especially frustrating in studies of plant organ growth and development, wherein the defining events that establish final-stage organ morphology may occur much earlier in ontogeny. In a new study published in *Genome Biology* from the Dirk Inzé laboratory [2], a novel strategy helps to resolve this conundrum by employing multi-scale, high-throughput phenotyping at multiple stages of leaf development and elongation. The resulting phenomic data are then correlated with transcriptomic analyses of the mitotically active cells that contribute to expansive leaf growth.

## Genome-wide analyses of leaf development: where are all the candidate genes?

Leaf organogenesis comprises three main stages (reviewed in [3, 4]). First, leaf initial cells are recruited from the shoot apical meristem periphery to form a leaf primordium. In stage two the main morphological domains of the leaf are differentiated and the young primordium assumes its basic shape. Finally in stage three, fine-scale differentiation and expansive growth transforms the young primordium into a mature leaf. In grasses and many other plant species, leaf development is basipetal (from tip to base). Fate mapping and analyses of cell division foci in maize have clearly illustrated that, during the later stage of leaf development, expansive growth and elongation occur predominately within the leaf base [3]. Leaves are quite amenable to mature-stage phenomic analyses, but the inaccessibility of young leaf primordia makes analyses of ontogenic events challenging. This is especially true in grasses such as maize, in which the young leaf primordia are enclosed within multiple whorls of older leaves [5]. For this reason, most high-throughput phenomic studies have focused on mature, adult-stage leaves.

Previous mutant screens have identified a number of interesting ‘master regulator’ genes required for leaf development, for which loss of function mutations result in extreme mutant phenotypes (reviewed in [4]). However, the relevance of these candidate genes in the regulation of leaf size and shape diversity in populations harboring widespread allelic variation has not been easy to decipher. One such genome-wide association study (GWAS) of a population of 5000 maize recombinant inbred lines (RILs) derived from 25 diverse inbred parents identified no candidate master regulator genes that were associated with leaf length or width [6]. A second study used the same population of RILs and examined the same mature-stage leaf phenotypes, but supplemented the genomic single nucleotide polymorphisms (SNPs) used to identify allele–phenotype associations with transcriptomic SNPs derived from RNA sequencing (RNA-seq) of seedling shoot apices [7]. Although this GWAS did identify candidate master regulator genes that were associated with leaf size, the vast majority of trait-associated SNPs were found in non-genic regions. These studies raise several intriguing questions. How much does allelic variation within leaf development master regulator genes actually contribute to leaf phenotypic variation in natural populations? Are these results perhaps merely reflective of the methodological peculiarities of GWAS? Alternatively, will focused and in-depth phenotypic analyses that include earlier ontogenic stages of leaf development help clarify this question?

## A novel approach to the phenomic analysis of leaf development

In their high-throughput phenotypic study of maize leaf development, Baute et al. [2] used a different approach. They employed careful phenotyping during multiple ontogenic stages of leaf elongation and maturation, coupled with focused RNA-seq analyses of the leaf division zone (DZ), a microscopic region of high mitotic index near the base of the emerging leaf (described in [8]). They showed that the size of the DZ is correlated with multiple mature-stage leaf size traits. In this way, they were able to link the macroscopic phenotypes of the developing maize leaf with specific transcriptomic profiles from the microscopic DZ, which functions earlier in leaf ontogeny.

In their study, they used an F12 population of 103 RILs derived from the maize inbred parental lines B73 and H99, and obtained detailed phenotypic analyses of multiple traits that correlated with the final size of leaf 4. Specifically, they assayed the mature-stage phenotypes of leaf 4: leaf length, width, area, and weight. The phenotypic data were complemented by high-throughput phenotypic analyses of the dynamics of leaf 4 growth over developmental time. These traits included the DZ size of leaf 4 at steady-state growth, time of leaf emergence from the whorl, leaf elongation rate (LER), time point of maximal LER, time point when leaf 4 reached its final length, and leaf elongation duration. Taken together, these data provide estimates of the length of time needed to fully expand leaf 4. An important conclusion stemming from this work is that final leaf size is strongly correlated with both rate and duration of leaf elongation, and more interestingly, that the mechanisms controlling the rate and duration of elongation are only partially shared.

## Correlating transcriptomics and phenomics

Baute et al. [2] then extended their study to perform transcriptomic analyses of the DZ in their RIL population to identify transcripts implicated in affecting final leaf size. The strategy to focus their transcriptomic analyses on the DZ, a region of high mitotic index, is supported by previous studies wherein mutants affecting final leaf size had profound effects on cell number as opposed to cell size (reviewed in [9]). By sampling a discrete 0.5 cm region of the DZ while leaf 4 was still in the steady-state phase of growth, the authors increased their chances of detecting differentially expressed candidate genes.

In the past decade, as RNA-seq has emerged at the forefront of transcriptomic research, the consensus is that these experiments are especially powerful when sampling is focused on discrete cells and tissues of isogenic plants, and during specific ontogenic time points [10]. Baute et al. [2] found that the top 1 % of DZ transcripts showing significant correlations with leaf phenotypes were enriched for gene categories that included ‘regulation of transcription’, ‘hormone metabolism’, ‘cell wall synthesis and degradation’, ‘cellular transport’, and ‘protein synthesis, degradation and modification’. Moreover, these DZ RNA-seq analyses identified a number of leaf size-correlated maize transcripts with homology to *Arabidopsis* genes implicated in cell division and/or leaf growth. These included maize homologs of growth-regulating factors 1 (GRF1) and 2 (GRF2), the TIR1/AFB auxin receptor protein, the brassinosteroid receptor DWARF1/DIMINUTO, several cytokinin-binding receptors (histidine kinases), and a number of genes predicted to function during protein ubiquitination (*BIG BROTHER, DA1*, *PC10*, and *SAMBA*) that show defects in mature leaf size when mutated in *Arabidopsis*.

## What’s next?

This study has shown the power of phenomic analyses that focus on a discrete organ, in this case leaf 4, during an ontogenetic window encompassing leaf elongation to leaf maturity. The exciting discovery that the size of the DZ is correlated with mature-stage leaf 4 traits enabled the authors to extend their correlation analyses to include gene expression within the DZ. By focusing their phenomic and transcriptomic analyses on a discrete organ and developmental stage, the authors reduced the distracting background noise that can often contaminate a less focused analysis. This study has generated a rich and precise transcriptomic data set, and a model for leaf size determination that implicates separate pathways regulating the rate and duration of leaf elongation. This model is eminently testable. Genetic analyses of the differentially expressed gene transcripts identified in this study can now be combined with the focused and multidimensional phenotypic analyses described by Baute et al. [2] to advance this next generation of plant phenomics. The ultimate goal is to extend high-throughput phenomic analyses to include the entire ontogeny of leaf development.

## Abbreviations

DZ: Division zone
GRF: Growth-regulating factor
GWAS: Genome-wide association study
LER: Leaf elongation rate
RIL: Recombinant inbred line
RNA-seq: RNA sequencing
SNP: Single nucleotide polymorphism
